# Optimizing Rooting and Growth of *Salvia rosmarinus* Cuttings in Soilless Systems Affected by Growth Regulators

**DOI:** 10.3390/plants14142210

**Published:** 2025-07-17

**Authors:** Georgios Lykokanellos, Ioannis Lagogiannis, Aglaia Liopa-Tsakalidi, Sofia Anna Barla, Georgios Salachas

**Affiliations:** 1 Department of Agriculture, University of Patras, Nea Ktiria, 30200 Mesologhi, Greece; aliopa@upatras.gr (A.L.-T.); sa_barla@yahoo.gr (S.A.B.); gsal@upatras.gr (G.S.); 2 Plant Protection Division of Patras, ELGO-Demeter, N.E.O. & Amerikis Ave., 26444 Patras, Greece; ilagogiannis@elgo.gr

**Keywords:** rosemary propagation, hydroponics, mist system, aeroponics, float system, growth retardants, daminozide, paclobutrazol, rooting hormones

## Abstract

This study investigated how propagation systems, growth regulators, and hormone formulations interactively affect the rooting and subsequent growth of rosemary (*Salvia rosmarinus* Spenn) cuttings. A three factorial (3 × 2 × 7) experiment was conducted under a fully controlled greenhouse environment, incorporating three soilless propagation systems (mist, float, aeroponics), two rooting hormone formulations (powder and gel-based IBA), and two growth regulators (paclobutrazol and daminozide) at three concentrations each. Significant differences (*p* < 0.001) were found in shoot height, root length, and number of lateral roots. The float system combined with powder hormone and no retardants achieved the highest shoot height (mean = 16.7 cm), while aeroponics with powder hormone and daminozide 1000 ppm promoted the greatest root branching (mean = 12.2 lateral roots per cutting). Root length was maximized (mean = 15.9 cm) under float systems with daminozide 1000 ppm. High doses of both growth regulators negatively affected all parameters across systems. Post-transplantation monitoring confirmed that cuttings from float and mist systems treated with powder hormone and low or no growth retardants exhibited superior establishment and net growth over 60 days. These findings demonstrate the critical importance of pairing hormone type, regulator concentration, and propagation system, providing actionable protocols for nursery managers aiming to enhance *Salvia rosmarinus* propagation in commercial practice.

## 1. Introduction

Medicinal and aromatic plants are of increasing agronomic, pharmaceutical, and commercial importance, playing a pivotal role in the evolution of sustainable agriculture [1]. Among them, *Rosmarinus officinalis* L., commonly known as rosemary, belongs to the *Lamiaceae* family. The genus *Rosmarinus* has been merged into the genus *Salvia* in a recent phylogenetic analysis. This means that *Rosmarinus officinalis* is no longer the correct name of the species studied. Since the name *Salvia officinalis* was already taken when the merger was decided, this species needed a new specific epithet in *Salvia*, so it is now known under the name *Salvia rosmarinus* [2,3,4,5]. Rosemary (*Rosmarinus officinalis* L., syn. *Salvia rosmarinus* Spenn), is a Mediterranean shrub, widely used for its essential oils, bioactive compounds, and antioxidant capacity, with applications ranging from culinary to cosmeceutical and ornamental sectors [6,7], as well as their postharvest and floriculture applications in the ornamental industry [8].

However, despite its economic value, rosemary propagation in commercial horticulture faces several significant challenges, including low rooting success rates, variability in rooting uniformity, and inconsistencies in subsequent plant growth, often influenced by propagation techniques and environmental conditions [9,10,11]. These issues can lead to economic losses and operational inefficiencies in commercial nurseries.

*Salvia rosmarinus*, valued for its aromatic and medicinal properties, demands efficient propagation methods for commercial production. Global production of rosemary essential oil alone exceeds 2000 tons annually, with major export markets in Europe and North America. While previous studies highlighted the advantages of soilless propagation systems—such as float stations and aeroponics—in enhancing rooting efficiency and quality, limited attention has been given to how these methods interact with chemical modulators such as rooting hormones (auxins) and growth retardants. Existing literature has frequently investigated these components in isolation, neglecting the complexity and the potential synergistic or antagonistic interactions that can profoundly impact nursery practices.

Recent studies indicate that rooting hormones and growth retardants can significantly influence plant morphology, but their effectiveness varies widely with propagation environments [12,13,14]. For instance, auxin-based rooting hormones such as indole-3-butyric acid (IBA) have shown varying efficacy depending on formulation type (powder vs. gel), primarily due to differences in adherence, absorption rates, and hormonal stability under different humidity and oxygenation conditions [15,16], often mediated by phenolic oxidation that limits auxin availability and rooting response [17]. Growth retardants, such as daminozide and paclobutrazol, are routinely applied to control vegetative growth, yet their interactions with different soilless systems and hormone formulations remain poorly characterized, often resulting in inconsistent propagation outcomes [18,19,20].

Although several independent studies have addressed these factors in isolation, very few have evaluated their interactive effects in a factorial manner. Sharma and Bhatia [21] documented the advantages of intermittent mist systems for ornamental propagation but did not include hormonal regulators. Savvas and Gruda [9], as well as Lakhiar et al. [10], emphasized the aeration benefits of aeroponics, yet without exploring its integration with hormone-retardant interactions. Similarly, Blythe [14] and Jain and Sharma [15] highlighted variability in auxin response depending on formulation and environmental factors, while Runkle and Blanchard [22] and Warner and Lohr [23] demonstrated that high doses of paclobutrazol may induce adverse physiological effects in ornamental crops, including reduced root initiation and chlorosis symptoms [24]. These gaps underscore the need for a robust factorial framework capable of elucidating such complex interactions in rosemary propagation.

In the Mediterranean context, *Salvia rosmarinus* has been actively studied in relation to propagation methods optimized for arid environments. Koukounaras et al. [25] reported variable rooting performance when rosemary cuttings were subjected to different substrate moisture profiles in semi-hydroponic systems under controlled Greek greenhouse conditions. Similarly, Tsouvaltzis and Korkovelos [26] investigated the comparative efficacy of powder versus gel auxin formulations in Mediterranean aromatic species and found context-dependent variations in rooting efficacy and early shoot vigor. Additional evidence from Grigoriadou et al. [27] supports the idea that microclimatic control in mist and float systems may enhance cutting viability, but only when hormonal and substrate compatibility is carefully calibrated. These findings reinforce the importance of locally adapted factorial approaches in aromatic plant propagation research.

This study aims to systematically explore these interactions through a comprehensive factorial design, bridging significant knowledge gaps and offering robust guidelines for nursery management practices. Given the increasing global demand for sustainable propagation methods, enhancing the efficiency and consistency of rosemary cultivation directly supports commercial viability and environmental sustainability.

In recent years, soilless cultivation systems such as hydroponics, mist, and aeroponics have emerged as highly effective propagation technologies, offering precise control over nutrient, water, and oxygen dynamics, which are critical in regulating root system architecture [25,28,29]. Aeroponics, in particular, offers a water-efficient, oxygen-rich environment conducive to rapid root formation and has been successfully used in both research and commercial production [30,31]. Float systems, though more traditional, remain efficient in root elongation [32], especially in herbaceous species such as rosemary [33], while mist systems offer balanced moisture and aeration conditions suitable for cuttings.

These propagation technologies are aligned with established hydroponic and aeroponic standards in controlled environments [34,35], and reflect best practices in modern protected cultivation systems [36]. The choice of these three systems—mist, float, and aeroponics—is justified by their distinct capabilities to regulate moisture, oxygen, and nutrient availability, critical factors influencing rooting success and plant development. Mist systems offer controlled intermittent moisture delivery beneficial for balanced growth; float systems provide constant nutrient availability and humidity, favoring root elongation; and aeroponics maximizes oxygen availability, supporting rapid and extensive root branching. The evaluation of powder and gel hormone formulations addresses differences in absorption efficiency and stability under varying humidity and oxygen conditions, essential for optimizing rooting protocols. Furthermore, daminozide and paclobutrazol were chosen due to their widespread commercial usage and known dose-dependent effects on plant morphogenesis, with potential for both beneficial and detrimental impacts depending on propagation environment and dosage [37,38,39], as also documented in recent work showing phytotoxic responses at elevated concentrations [40].

Despite these advances, the production of healthy, uniform propagating material remains a bottleneck in aromatic plant production [41,42]. One key challenge is the optimization of hormonal treatments, including rooting hormones (auxins) and growth retardants, to modulate shoot and root architecture. However, existing research often overlooks the complex interactions between propagation methods and chemical modulators, leaving significant knowledge gaps and resulting in inconsistent nursery practices.

Auxins like indole-3-butyric acid (IBA) are widely used in powder or gel formulations, each with distinct absorption dynamics depending on environmental context and potential interactions with phenolic oxidation during rooting [1,14]. While some studies have shown positive growth effects from moderate applications of growth retardants, others reported reduced propagation efficiency due to unpredictable interactions with auxins under specific propagation systems.

Similarly, growth retardants such as daminozide and paclobutrazol are commonly employed to regulate stem elongation, enhance compactness, or balance shoot/root ratios [18,19]. However, the interaction between propagation system, hormone formulation, and retardant dose has rarely been studied in a fully factorial, statistically robust framework, which is essential for accurately detecting interaction effects in plant morphological traits [20]. This analytical framework was implemented through a three-way ANOVA following established agricultural statistical protocols [43]. Our own results confirmed that not only do all individual factors significantly affect plant morphology (*p* < 0.001), but also their two-way and three-way interactions are critical for rooting success, shoot height, and lateral root formation. For example, powder hormones significantly increased shoot height and root length in float and mist systems, while gel hormones performed better in aeroponics, particularly under moderate retardant levels. Daminozide at 1000 ppm enhanced root branching and post-transplant vigor, whereas high doses of both retardants inhibited growth, particularly in aeroponics.

Therefore, the current study was designed to explore these complex relationships and optimize propagation protocols for *Salvia rosmarinus* using advanced soilless systems, rooting hormones, and growth regulators. To our knowledge, this is the first comprehensive factorial analysis integrating three propagation variables simultaneously in *Salvia rosmarinus*. The specific research objectives were: (1) to evaluate the interactive effects of propagation systems (mist, float, aeroponics), hormone formulations (powder, gel), and growth retardants (daminozide, paclobutrazol) on rosemary morphology and rooting efficiency; (2) to identify optimal treatment combinations for enhanced nursery performance and post-transplant vigor. Our hypotheses are that (a) significant interactions among propagation systems, hormones, and retardants will exist and (b) optimal combinations will be system-specific, thus requiring tailored propagation protocols. The experiment involved 42 treatments (3 systems × 2 hormones × 7 retardant levels including control) and was conducted in a fully controlled greenhouse facility. Evaluated parameters included plant height, root length, root branching, and post-transplant field adaptation, integrating statistical rigor with agronomic relevance.

## 2. Materials and Methods

The present study was carried out between March and June 2023 in a fully automated polyethylene-covered greenhouse at the Department of Agriculture, University of Patras. This facility is equipped with a high-precision climate control system capable of regulating temperature, humidity, and photoperiod according to predefined crop requirements. Temperature was maintained consistently at 25 ± 2 °C during the day and 18 ± 2 °C at night. Relative humidity was kept at 70 ± 5%, and photosynthetic photon flux density (PPFD) was adjusted to 150 μmol·m^−2^·s^−1^ using full-spectrum LED grow lights. A 16 h photoperiod (06:00–22:00) was applied uniformly throughout the trial. Ventilation, CO_2_ levels (ambient), and misting systems were synchronized with a programmable logic controller (PLC) for environmental consistency. Sensors were deployed at plant canopy and root level, and data were logged hourly. All environmental parameters were continuously monitored using digital sensors and logged via an integrated data management platform.

Uniform and pathogen-free cuttings of *Salvia rosmarinus* were collected from lateral shoots of healthy stock plants previously screened via ELISA assays to ensure sanitary status. The selected cuttings, measuring 5 to 8 cm in length, were prepared under sterile conditions and subjected to rooting treatments designed to evaluate the combined effects of hormone formulation, growth retardants, and propagation system.

The experimental design was a fully randomized three-factor factorial arrangement comprising 42 distinct treatment combinations. These combinations arose from three propagation systems (mist, float, and aeroponics), two rooting hormone formulations (powder and gel), and seven growth retardant levels, including an untreated control. Both growth retardants were prepared using commercial formulations. Daminozide, consisting of ALAR 85 SG (85.14% *w*/*w* active ingredient, K+N Efthymiadis ABEE) [44], was dissolved in distilled water at concentrations of 1.2, 3.0, and 6.0 g·L^−1^ to achieve application rates of 1000, 2500, and 5000 ppm, respectively. Paclobutrazol (Bonzi 4 SC, 0.4% w/v a.i., Syngenta) was diluted at 0.25, 0.5, and 1.25 mL·L^−1^ to obtain final concentrations of 1, 2, and 5 ppm, respectively. The solutions were stirred for 10 min and used fresh within 2 h to avoid degradation. The powder hormone used was a commercial IBA-based formulation containing 0.2% indole-3-butyric acid and 99.8% inert carrier (RootOn A 0.2 DP). The gel hormone consisted of IBA combined with vitamin B1 (thiamine hydrochloride) in a viscous matrix (Root!T formulation).

For each treatment, cuttings were first immersed in the appropriate growth retardant solution for 60 s using sterile polypropylene trays. Hormone application followed immediately, with the basal 1–1.5 cm of each stem dipped into either powder or gel formulation. Powder was applied by direct contact with sterile gloves, and gel with sterile cotton swabs. Application was carried out under a laminar flow hood to ensure aseptic conditions. Thirty cuttings were used per treatment, yielding more than 1200 experimental units (Table 1). Controls received the same handling but without growth regulator exposure.

**Figure 1:**
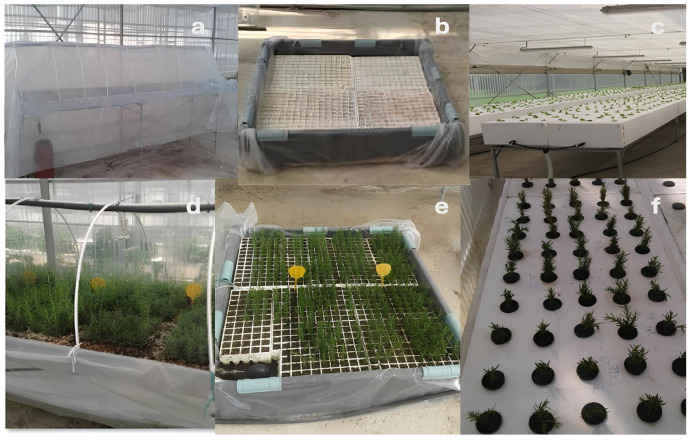
Representative images of the three propagation systems used in the experiment: (**a**) mist, (**b**) float, and (**c**) aeroponic. Panels (**d**–**f**) depict the respective systems during the rooting phase, showing inserted rosemary cuttings under active propagation conditions.

The three propagation systems were assembled and operated based on the Department’s standardized protocols. The mist system (Figure 1a) consisted of a bench-mounted intermittent misting mechanism that delivered fine droplets to the cuttings at controlled intervals. The misting system used 0.2 mm fog nozzles delivering droplets of 22–80 µm. Misting operated on a 10 s per 15 min interval (07:00–20:00), controlled by an Irritrol Junior MAX 4-station electronic timer (Toro Company, Riverside, CA, USA) connected to solenoids and humidity sensors. The rooting substrate consisted of a 1:1:1 mix of steam-sterilized enriched peat (50% blonde and 50% black peat, pH ≈ 6.0, EC ≈ 45 mS/m), perlite (Perloflor FINE, Perlite Italiana S.r.l., Corsico, Italy), and vermiculite (light-grade layer, 1 mm thick) with slow-release micronutrients (Florand 12-14-24 + TE, Compo Expert GmbH, Münster, Germany). Black polyethylene sheeting was used to enclose the rooting frame to stabilize humidity at ~90%. The system was supplemented with an aeration module driven by a submersible pump to prevent stagnation and anoxia.

The float system (Figure 1b) comprised rectangular wooden tanks measuring 1.2 m by 1.2 m and 0.3 m in depth. These were internally lined with black polyethylene sheets to prevent light exposure and to retain the nutrient solution. Cutting trays floated on the surface, allowing continuous contact between the stem base and the liquid medium while ensuring passive oxygenation. The nutrient solution was based on a half-strength Hoagland formulation, continuously aerated with a dual-outlet AirPump AR-8500 (Resun, Shenzhen, China) and replaced every 7 days. A water refill system with float valve ensured constant volume. Solution pH and EC were monitored twice per week using a handheld pH meter (model HI98107, Hanna Instruments, Woonsocket, RI, USA) and EC meter (model Cond 3310, WTW, Weilheim, Germany), targeting pH 5.8 ± 0.1 and EC 1.4 ± 0.2 mS/cm. A tunnel structure with polyethylene cover retained RH > 85%.

In the aeroponic system (Figure 1c), the roots of the suspended cuttings were exposed to fine nutrient mist delivered by calibrated nozzles under moderate pressure. The aeroponic units were constructed to a height of 30 cm to accommodate root elongation without contact with the base. Mist nozzles with 0.3 mm orifice sprayed nutrient mist every 10 min for 5 s, controlled by solenoid valves and a programmable timer. The chamber was covered with a custom-fit Styrofoam lid to hold the cuttings and ensure darkness. An additional polyethylene tunnel enclosed the entire structure to stabilize relative humidity at >90%. Aeroponics and hydroponics used full nutrient solutions, which provide all essential nutrients except carbon. Only 12 of the 16 essential nutrients needed to be added, typically via water-soluble salts or chelated compounds. The final nutrient solution was a diluted aqueous mixture containing inorganic ions and soluble compounds. Environmental and misting parameters were controlled through integrated relays and monitored using embedded sensors. The nutrient solution used for irrigation had the following macronutrient composition (mM): K^+^, 6.5; Ca^2+^, 3; Mg^2+^, 0.9; NO_3−_, 6.75; NH^4+^, 0.36; H_2_PO^4−^, 1.6, and micronutrient (μM): Fe^2+^, 30; Mn^2+^, 5; Zn^2+^, 4; Cu^2+^, 0.75; B, 30; Mo, 0.53. pH was adjusted to 5.6 by the use of HNO_3−_ and the electrical conductivity was kept at 1.70 dS/m. The temperature of the nutrient solution was kept at 20 °C automatically. Preparation of the nutrient solution, adjustment of pH, and electrical conductivity were controlled electronically.

Throughout the rooting phase (Figure 1d–f), environmental parameters were recorded daily, and the physical condition of the cuttings was inspected. Measurements were taken at day 45 after planting. Shoot height was measured using a digital caliper (Mitutoyo Europe GmbH, Neuss, Germany, ±0.1 mm accuracy) from the stem base to apical meristem. Root length was recorded as the maximum elongation of the principal root using a flexible metric tape. Root branching was quantified by counting visible lateral roots > 0.5 cm under a Leica EZ4 stereomicroscope (Leica Microsystems GmbH, Wetzlar, Germany) (10× magnification). Measurements were taken of 10 randomly selected cuttings per treatment. Following successful rooting, representative samples from each treatment were transplanted into uniform soil-filled containers. These plants were monitored under open-field conditions for a period of 60 days, during which data on transplant shock, survival rate, and net shoot elongation were collected to evaluate post-propagation vigor.

All measured parameters—shoot height, root length and number of lateral roots—were subjected to three-way analysis of variance (ANOVA) to determine the effects of individual factors and their interactions. Where significant differences were detected, Tukey’s HSD test was applied to identify statistically homogeneous groups. Interaction effects were further explored using visual interaction plots, and the full statistical analysis was conducted using Python (version 3.10) (SciPy, v1.11.4, Statsmodels, v0.14.0) and IBM SPSS software (version 29.0), ensuring rigorous quantitative assessment of the experimental outcomes.

## 3. Results

**Figure 2:**
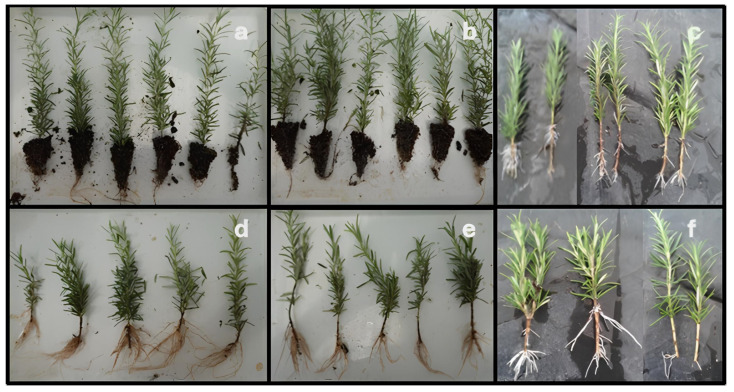
Representative image of rooted *Salvia rosmarinus* cuttings at the rooting stage under various propagation systems. (**a**–**c**) Powder hormone; (**d**–**f**) gel-type hormone).

This section presents the effects of propagation system, growth retardant, and hormone formulation on key morphological traits of *Salvia rosmarinus* cuttings. All parameters were evaluated through a three-way factorial design, and statistically significant differences were confirmed through ANOVA and post hoc Tukey HSD testing. The analysis of variance (ANOVA) indicated that all main effects and all possible interactions—including two-way and the three-way interaction—were statistically significant (*p* < 0.001), denoting a highly complex, multivariate control over shoot elongation in rosemary cuttings. The interactions among factors are discussed in the context of propagation efficiency, physiological response, and practical implications for nursery production. Representative rooting outcomes under the three systems are depicted in Figure 2, illustrating the morphological appearance of rooted *Salvia rosmarinus* cuttings prior to data collection. These visual references complement the quantitative analyses that follow (Figure 2).

### 3.1. Shoot Height

Shoot height was significantly influenced by all main factors and interactions. The cultivation system exerted the strongest effect, with float systems yielding the tallest shoots. Growth retardants, particularly paclobutrazol at higher concentrations, markedly suppressed shoot elongation. Hormone formulation also affected results, with powder outperforming gel in float and mist systems. The propagation system emerged as the most influential factor on plant height (F = 511.92). Plants propagated under the float system consistently reached the highest shoot heights, likely due to optimal root zone aeration, passive nutrient uptake, and reduced mechanical stress on developing roots. Mist propagation followed, providing intermediate shoot elongation, whereas aeroponics yielded the shortest plants. This may relate to transient water stress or mechanical resistance from fine droplet formation in aeroponic conditions. Growth retardants significantly modulated elongation. Paclobutrazol (1–2 ppm) and daminozide (1000–5000 ppm) both had an inhibitory effect on stem extension, consistent with their known role in gibberellin biosynthesis inhibition. At high concentrations (5000 ppm), both compounds caused marked reduction in height across all systems. However, at lower doses (especially 1000 ppm daminozide), some growth stimulation was observed depending on the system and hormone interaction. The effect of hormone formulation (gel vs. powder) was statistically significant (F = 36.11, *p* < 0.001). Powder formulations supported greater elongation, potentially due to more effective contact at the basal cutting surface, controlled release, and minimized dilution in aqueous systems. This trend was particularly prominent in float and mist systems. Gel formulations, although more manageable during application, may lead to slower or less uniform hormone absorption (Table 2).

Three-way ANOVA revealed that all main factors—system, retardant, and hormone formulation—had significant effects on shoot height (*p* < 0.001), along with all two-way and the three-way interaction terms. The cultivation system exerted the most dominant effect (F = 511.92), with float systems producing the tallest plants, followed by mist, and significantly shorter plants under aeroponics. Growth retardants showed strong inhibitory effects, especially at higher concentrations. Notably, daminozide at 5000 ppm and paclobutrazol at 2–5 ppm significantly suppressed elongation. The hormone formulation had a significant but moderate influence (F = 36.11), with powder hormones promoting greater elongation in float and mist systems.

The system × hormone × retardant interaction was highly significant (F = 3.31) (*p* < 0.001), indicating that the effect of hormones or retardants cannot be evaluated in isolation: no single factor could predict plant height without considering the others. For example, the superior shoot elongation observed in float systems with powder hormone and no retardant can be attributed to the continuous nutrient saturation and stable humidity, enhancing auxin uptake and translocation. In contrast, gel formulations in aeroponics resulted in lower shoot height, possibly due to limited adhesion and uneven hormone distribution in the high-oxygen mist environment. The presence of high-dose paclobutrazol (5 ppm) further exacerbated the inhibition of elongation, likely due to suppressed gibberellin biosynthesis, which is particularly sensitive in highly aerated systems like aeroponics. These findings emphasize the need for carefully matched inputs: powder IBA worked optimally under high-humidity, nutrient-rich substrates such as float beds, whereas gel hormones did not provide consistent delivery in mist-dominated or oxygen-saturated systems. From a commercial propagation standpoint, nursery operators should avoid combining high doses of growth retardants with aeroponic systems unless compact growth is the target.

The Tukey HSD test (Tukey HSD, *p* < 0.05) revealed statistically distinct groupings among treatment combinations. The results validate that hormonal regulation and environmental conditions are not interchangeable across systems and must be co-optimized. Post hoc comparisons identified the combination of float system + powder hormone + no retardant as the highest-performing treatment. In contrast, aeroponic systems with gel hormone and high retardant levels were consistently the lowest-performing groups. This supports the recommendation that commercial propagation aiming for height elongation should prioritize float systems with powder-based auxins and avoid inhibitory retardant levels. Powder-based hormones were more effective in float and mist systems, where they could adhere to the stem base and promote uniform auxin availability. In aeroponic systems, gel formulations underperformed, possibly due to poor interaction with the highly oxygenated, humid environment where gels could dilute or wash off. The response to growth retardants varied significantly by propagation system. For instance, aeroponic plants were highly sensitive to even low doses, showing substantial height reduction. Mist and float systems mitigated these effects, especially under moderate retardant concentrations (e.g., daminozide 1000 ppm). The three-way interaction was statistically significant (F = 3.31, *p* < 0.001), confirming that optimal shoot elongation depends on the specific combination of hormone type, retardant level, and propagation environment. For example, float + powder + control consistently outperformed other combinations, while aeroponics + gel + high retardant resulted in the poorest growth metrics (Table 3).

**Figure 3:**
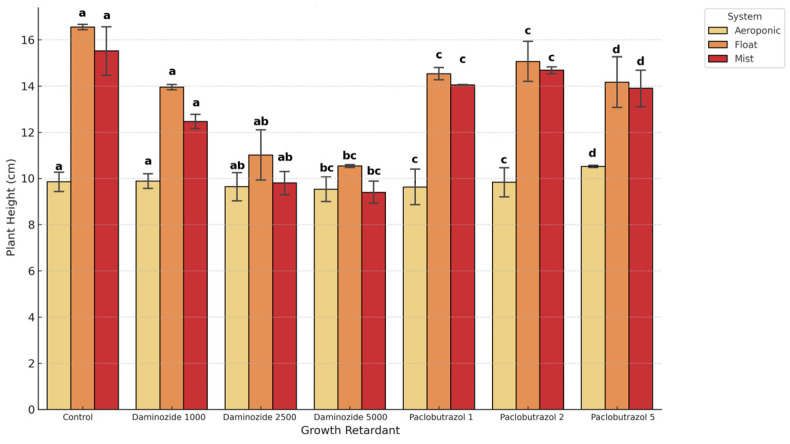
Interaction effects of propagation system, and growth retardant on shoot height (cm) of *Salvia rosmarinus* cuttings. Each point represents the mean ± SD of 30 replicates per treatment. Different lowercase letters (a–d) above the bars indicate statistically significant differences among treatment means according to Tukey’s HSD test at *p* < 0.05. Bars that share at least one common letter do not differ significantly.

The interaction plot (Figure 3) reveals clearly non-parallel trends, illustrating how plant height is differentially affected by growth retardants depending on the propagation system and auxin formulation. These visual patterns—supported by a statistically significant three-way interaction (*p* < 0.001)—indicate that optimal responses were highly context-dependent, with no single factor acting in isolation. Specifically, maximum shoot elongation was recorded in float systems treated with powder-based auxins and no growth retardants. In contrast, aeroponic systems combined with gel formulations and high concentrations of paclobutrazol resulted in substantial suppression of plant height.

### 3.2. Root Length

Root elongation was likewise affected by all primary and interaction factors. Float systems produced the longest roots, and moderate levels of daminozide (1000 ppm) enhanced root growth. The propagation system again exerted the most dominant effect on root length (F = 226.62). The float system consistently promoted the longest root elongation, likely due to the stable water film and minimal mechanical resistance, allowing for free axial development. The mist system ranked second, where intermittent moisture pulses permitted substantial but slightly restricted growth. Aeroponics consistently resulted in shorter roots, possibly due to transient root desiccation between mist cycles and increased oxygen levels, which may signal early root maturation and reduced elongation. The presence and concentration of growth retardants had a profound impact (F = 65.34). Daminozide at 1000 ppm notably promoted root elongation, particularly in float and mist systems. However, at higher concentrations (2500–5000 ppm) and with paclobutrazol (1–2 ppm), a marked suppression of root growth was observed, especially under aeroponic conditions. These results are aligned with the known inhibitory action of these compounds on cell elongation and division via gibberellin pathway disruption. Though its individual effect was less pronounced (F = 5.06, *p* = 0.0247), the hormone formulation significantly influenced root elongation depending on context. Gel-based hormones performed better in aeroponics, likely due to improved adherence and slower dissipation under high-humidity, oxygen-rich conditions. In contrast, powder formulations outperformed gels in float and mist systems, where they were better retained and activated by consistent contact with moist substrates. Root elongation under gel vs. powder hormones depended on the system. In aeroponics, gel clearly yielded better root lengths, suggesting a superior performance in moisture-deficient or droplet-dependent environments. In contrast, in float systems, powder outperformed gel, likely due to better activation through immersion contact. The system-dependent effect of growth retardants was pronounced. While daminozide 1000 ppm promoted elongation in float and mist, the same concentration did not overcome the inhibitory environment of aeroponics. Aeroponic systems amplified the inhibitory effects of high-retardant concentrations, possibly due to limited buffering capacity or faster chemical uptake due to thin root layers. This interaction was statistically highly significant (F = 4.26, *p* < 0.001), confirming that no single factor predicts root length independently (Table 4).

Root elongation was also significantly affected by all tested factors and their interactions. The propagation system had the highest influence (F = 226.62), with float systems producing the longest roots, followed by mist and aeroponics. The effect of growth retardants was dose-dependent: daminozide at 1000 ppm promoted root elongation, while higher concentrations of both retardants suppressed growth, especially under aeroponics. Powder hormone formulations generally outperformed gel in mist and float systems, but gel showed marginally better performance in aeroponics.

Tukey HSD comparisons showed that float + powder + daminozide 1000 ppm resulted in the longest roots, whereas aeroponics + gel + paclobutrazol 5 ppm resulted in the shortest (Table 5). The data suggest that hydroponic propagation requires precise matching of hormonal and chemical inputs to the root environment. These findings suggest that the physiological state of cuttings under different hydroponic regimes modifies their hormonal responsiveness and elongation capacity.

**Figure 4:**
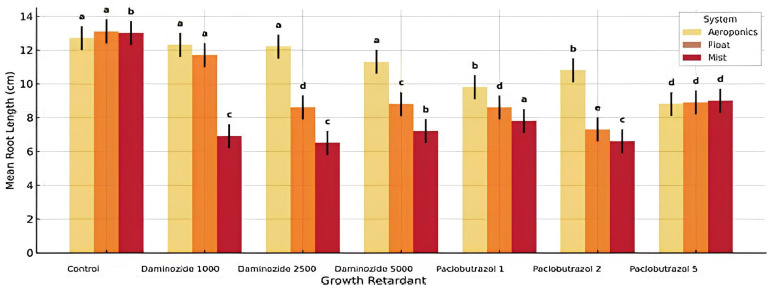
Interaction effects of propagation system, and growth retardant on root length (cm) of *Salvia rosmarinus*. Each point represents the mean ± SD of 30 replicates per treatment. Different lowercase letters above bars indicate statistically significant differences among treatment means according to Tukey’s HSD test at *p* < 0.05. Bars sharing the same letter are not significantly different.

Interaction plots (Figure 4) confirmed system-dependent responses to retardants and hormone type. The presence of non-parallel lines and crossover points illustrates that retardant effects are modulated differently within each system and hormone context. For example, root length under powder hormone sharply declined with increasing retardant concentration in aeroponics, while float and mist systems maintained better performance under similar chemical stress. Significant three-way interactions were observed (*p* < 0.001). Root elongation was significantly promoted in float systems with moderate daminozide levels (1000 ppm) and powder hormone application. Aeroponic setups showed decreased root length under higher paclobutrazol doses, especially with gel formulations. The three-way interaction (F = 4.26) was particularly informative, confirming that no single variable alone predicts optimal root length. Float systems enhanced auxin diffusion and oxygenation, supporting root cell expansion and elongation, especially in combination with powder-based IBA and moderate daminozide (1000 ppm). In contrast, aeroponic systems—despite their high oxygen availability—did not favor root length unless hormone uptake was optimized. The superior results under float + powder + daminozide 1000 can be explained by synergistic action: powder auxin provides slow, consistent release in moist conditions, while daminozide at this concentration may modulate gibberellin levels sufficiently to prevent excessive shoot elongation while favoring root formation. Under aeroponic conditions, high-pressure mist delivery may result in hormone wash-off or insufficient contact time, reducing efficacy. Practically, nurseries aiming for enhanced root systems should combine float beds with powder auxins and calibrated daminozide doses rather than rely solely on high-tech aeroponics.

### 3.3. Root Branching

The number of lateral roots per cutting showed a significant response to all treatments. Mist systems with powder hormone and daminozide 1000 ppm showed the highest branching (Table 6). Root branching was most influenced by the propagation system (F = 113.61), with the mist system promoting the highest number of lateral roots, followed by float and lastly aeroponics. This may be attributed to the cyclical wet/dry regime of misting, which induces moderate stress and oxygen-rich conditions favoring lateral root initiation. In contrast, aeroponics—despite its oxygen availability—possibly failed to maintain sufficient hormone contact, reducing branching frequency. Growth retardants exhibited a dose-dependent response. Lower concentrations of daminozide (1000 ppm) promoted lateral root formation, consistent with its known capacity to balance apical dominance and stimulate lateral organogenesis. At higher doses (2500–5000 ppm), retardants suppressed branching in all systems, particularly under aeroponic conditions. The hormone formulation had a statistically significant influence (F = 18.55). Powder formulations were superior in inducing root branching, likely due to higher localized auxin concentrations at the basal cutting site. This effect was most pronounced in mist and float systems. In aeroponics, both formulations performed poorly, though gel showed slightly better consistency due to improved adhesion.

The number of lateral roots per cutting was significantly influenced by all factors. ANOVA showed high significance for system (F = 113.61), hormone formulation (F = 18.55), and growth retardant concentration (F = 42.82). Mist systems induced the highest overall branching, particularly under powder hormone and daminozide 1000 ppm. Aeroponics also showed enhanced branching but only under moderate retardant concentrations (2 ppm). High doses of either retardant suppressed branching across all systems.

Powder hormone showed significantly higher branching in mist and float systems, while gel formulation underperformed across all systems except marginally in aeroponics. This may reflect reduced auxin transport efficiency from gel carriers under varying humidity and contact conditions. The inhibitory effects of retardants were amplified in aeroponics, where even moderate concentrations led to sharp reductions in branching. Conversely, mist and float systems buffered these effects, enabling branching even under daminozide 2500 ppm. The three-way interaction was significant (F = 3.98, *p* < 0.001), confirming that the effect of hormone formulation is conditional upon both retardant type and system environment. For instance, powder hormone combined with daminozide 1000 ppm in the mist system consistently produced the highest number of lateral roots. This optimal combination was nullified in aeroponics, demonstrating system dependency.

Tukey HSD comparisons showed that the highest number of lateral roots (Table 7) occurred in the mist system using powder hormone and daminozide 1000 ppm. In contrast, the lowest branching was recorded in aeroponics under gel hormone and paclobutrazol 5 ppm. This highlights that branching is not a universal outcome of aeration: excessive chemical suppression via growth retardants can override environmental advantages unless tightly controlled.

**Figure 5:**
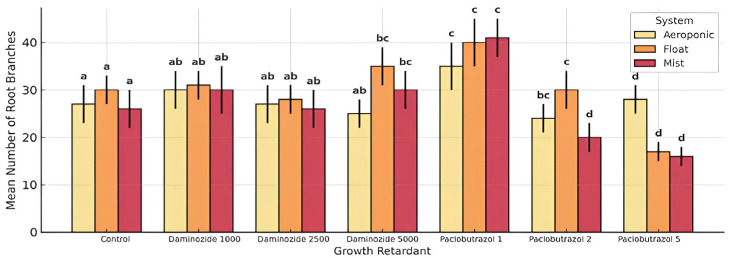
Interaction effects of propagation system, and growth retardant on the number of lateral roots per cutting. Each point represents the mean ± SD of 30 replicates per treatment. Different lowercase letters indicate statistically significant differences among treatments (*p* < 0.05) according to Tukey’s HSD test. Bars sharing at least one common letter (e.g., “a” and “ab”) are not significantly different from each other.

The interaction plot (Figure 5) reveals distinct patterns of lateral root formation, with crossing lines and divergent slopes confirming interaction effects. For example, powder hormone curves in mist and float systems follow an inverted-U trend, peaking at moderate retardant levels, while in aeroponics the same combinations drop off sharply. Interaction plots indicated distinct system-dependent modulation of branching responses. Significant three-way interactions were observed (*p* < 0.001). Mist systems, particularly when combined with powder IBA and daminozide at 1000 ppm, significantly enhanced lateral root formation. Aeroponics coupled with high retardant doses consistently resulted in lower branching, indicating potential phytotoxicity at elevated concentrations. The interaction between retardant and hormone formulation was highly significant, and the three-way interaction was also confirmed (F = 3.98), demonstrating that root branching is particularly sensitive to environmental inputs. These results highlight the importance of matching propagation inputs to the physiological context of each system. The mist system, by maintaining high and intermittent humidity with adequate drainage, supports not only main root elongation but also stimulates lateral meristem activity, especially when supplemented with powder auxin and low-to-moderate daminozide. The presence of powder-based IBA provides consistent auxin availability necessary for lateral root primordia initiation. In contrast, aeroponics showed improved branching only at intermediate retardant concentrations, possibly due to reduced auxin oxidation in highly aerated conditions. However, high doses of retardants likely caused hormonal imbalance, impairing lateral organogenesis. From a practical propagation perspective, optimizing for bushy, fibrous root systems—desirable for transplant stability—should involve mist propagation, powder auxins, and low-to-moderate daminozide applications.

The response curves for gel hormone treatments consistently displayed lower trajectories, indicating reduced efficacy across propagation environments.

### 3.4. Field Growth Performance (60 Days Post-Transplant)

**Figure 6:**
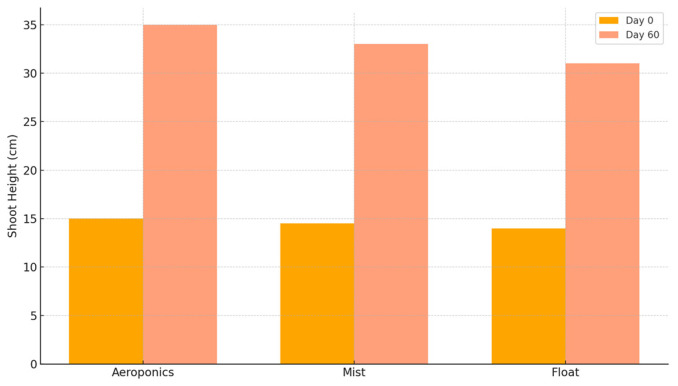
Post-transplant shoot elongation (cm) of *Salvia rosmarinus* cuttings under different propagation treatments. Measurements were taken 60 days after transplanting. Treatments that initially favored balanced shoot–root development (e.g., mist with powder hormone and daminozide 1000 ppm) demonstrated superior post-transplant vigor. Aeroponics with excessive retardants resulted in stunted elongation, highlighting lasting effects of early stress.

Following transplantation to soil containers, rosemary plants were evaluated for post-propagation vigor by monitoring shoot elongation over a 60-day period. All treatment groups demonstrated successful establishment. Net shoot growth ranged from 8.9 to 9.8 cm among treatments (Figure 6).

The float and mist systems achieved the highest net gains, particularly in untreated control groups treated with powder-based hormones. Aeroponic systems exhibited greater variability and a slight delay in establishment (Table 8). Overall, shoot height increase across all treatment combinations ranged from approximately 8.9 to 9.9 cm.

Due to the structure of the dataset, inferential statistics such as ANOVA were not applicable. Therefore, this section is based solely on descriptive trends and must be interpreted with caution. Future studies should expand the number of biological replicates in the post-transplant phase to enable statistical testing of establishment performance. The persistence of early-stage advantages into post-transplant growth supports the hypothesis that root system quality and shoot vigor at propagation stage directly affect subsequent establishment. Treatments that promoted robust root systems (e.g., mist + powder + daminozide 1000 ppm) maintained superior post-transplant performance. In contrast, treatments that caused initial suppression—especially those combining gel hormones and high doses of paclobutrazol—were less vigorous post-transplant. For nursery managers, this finding underscores the economic value of selecting combinations that balance rapid rooting with long-term vigor.

Despite the absence of statistical testing, visual inspection and treatment ranking revealed discernible trends such as that the highest net gains were observed in the mist and float systems, particularly under control treatments with powder hormone formulation, and the aeroponic-derived plants, while slightly trailing in performance, still exhibited satisfactory establishment, especially under gel hormone formulations and low-to-moderate retardant concentrations. No treatment group exhibited net growth suppression, indicating that all propagation strategies used in the controlled phase supported field adaptation. Notably, treatments that performed well during rooting also sustained superior vigor in the field.

Powder formulations may confer longer-lasting physiological priming, particularly in enhancing apical dominance and post-transplant vigor. Auxin retention during rooting likely facilitated continued meristematic activity after transplanting. The mist and float systems provided more buffered and stable rooting environments, which may result in more robust root systems and greater water/nutrient absorption efficiency post-transplant. Aeroponic systems, though effective for controlled growth, may result in root structures less adapted for abrupt transition to soil. Plants previously exposed to high concentrations of daminozide or paclobutrazol exhibited slightly lower growth rates in the field. These residual effects support the hypothesis of hormonal inertia, where biochemical suppression of gibberellin biosynthesis persists even after environmental transition. The full dataset of morphological parameters is provided in Supplementary Table S1.

## 4. Discussion

The present study delivers comprehensive insights into the interactive effects of propagation systems, growth regulators, and rooting hormone formulations on morphological and physiological responses of *Salvia rosmarinus* cuttings. These findings reinforce the importance of a tailored approach to propagation system selection and chemical input optimization, particularly under soilless cultivation frameworks.

The float system emerged as particularly effective in promoting shoot elongation and root development, corroborating findings that nutrient-rich aqueous environments enhance growth dynamics in herbs [32,33,45,46]. Mist systems, known for supporting uniform microclimatic conditions, achieved balanced shoot and root responses, aligning with documented benefits for intermittent moisture delivery and aeration [21,26,32,46]. Aeroponics strongly enhanced lateral root branching, attributable to optimal oxygenation and nutrient uptake efficiency—a trend consistent with current literature in aeroponic propagation of herbs and vegetables [9,10,20,24,30,34]. However, the comparatively lower shoot height under aeroponics highlights a physiological trade-off that must be considered when selecting systems for nursery production [22].

Growth regulators played a pivotal role in modifying plant morphology. Daminozide at 1000 ppm enhanced root length and branching, particularly under mist and float systems, consistent with studies noting moderate concentrations of daminozide improve rooting and transplant vigor [16,18,19,47,48,49,50]. In contrast, higher concentrations of daminozide and paclobutrazol inhibited shoot elongation and lateral root formation, especially under aeroponic conditions, corroborating data on phytotoxic effects at excessive doses [15,19,20,51,52]. Gallegos-Cedillo et al. [53] and Erwin and Warner [50] confirmed that excessive application of growth retardants induces physiological stress and suppresses meristematic activity in various horticultural species. These findings underscore the critical role of dose calibration in horticultural retardant applications [18,19].

Hormonal formulation was also a decisive factor. Powder-based IBA promoted greater shoot height and root elongation, likely due to enhanced adherence and absorption under high-humidity substrates such as float and mist [14,15,26,32,33,47]. Gel formulations showed marginal advantages exclusively under aeroponic systems, potentially due to improved auxin stability and localized hormone retention in high-oxygen microenvironments [15,54,55]. Nonetheless, the broader trend indicated that powder formulations consistently outperformed gel types across multiple systems [14,15,26].

Of particular interest is the effect of phenolic compounds on auxin stability during rooting—a factor often underestimated in propagation studies. Phenolic oxidation can significantly reduce auxin efficacy, as demonstrated by De Klerk et al. [17,56] and Kevers et al. [57] in their investigation of oxidative decarboxylation mechanisms. This phenomenon may partly explain the superior rooting performance observed in powder-based IBA treatments, as these formulations likely promote faster auxin uptake and reduce surface oxidation. In contrast, gel matrices may be more susceptible to phenolic compound interaction and hormone retention at the cutting site. This highlights the importance of selecting appropriate hormone types and formulations in relation to the propagation environment to minimize auxin degradation and maximize efficacy.

Furthermore, oxygen availability is increasingly recognized as a critical determinant in root zone performance. Ashraf and Foolad [24] highlight that oxygen saturation enhances root vigor and tissue differentiation—mechanisms particularly relevant in aeroponic environments, which demonstrated strong root branching in this study.

The float system also deserves special mention for its passive oxygenation capacity and nutrient delivery efficiency. Lee and Lee [46] and George et al. [33] reported that float-based hydroponics supported enhanced root biomass in herbs due to stable moisture and reduced mechanical stress.

Crucially, the statistically significant three-way interactions between system, hormone, and retardant confirm the non-linear, system-specific nature of plant responses. This aligns with modern understanding of genotype–environment–input interactions, which require factorial experimental designs for proper resolution [43,58,59]. The data suggest that no single factor operates independently, and propagation strategies must be developed contextually [33,59].

The current literature still lacks integrated, statistically rigorous studies that simultaneously evaluate environmental systems and chemical inputs. Although shoot elongation was monitored as a non-invasive proxy for field performance, dry weight data were not collected at 60 days to preserve material for ongoing secondary metabolite profiling. Future trials will incorporate destructive sampling to complement morphological assessments with total biomass data. Our findings provide a rare example of such integration and may serve as a model for future research [43,54]. Additionally, previous reviews on vegetative propagation of Mediterranean aromatic plants emphasize that rooting success depends not only on environmental conditions but also on hormonal compatibility and the genetic plasticity of each species [26,27,60].

Finally, the integration of propagation technologies within broader sustainability frameworks has become increasingly relevant. Recent studies stress the potential of hydroponics and aeroponics not only as propagation tools, but also as scalable, resource-efficient systems for commercial aromatic plant production [34,35,36,61]. These methods are particularly suited for urban and peri-urban horticulture, where land scarcity and water efficiency are critical [39,40,41]. In this context, vertical farming has emerged as a promising avenue for large-scale propagation and cultivation of *Salvia* *rosmarinus*, aligning with trends in controlled-environment agriculture (CEA) [62,63].

However, caution should be exercised regarding the over-application of chemical growth regulators in such systems. Excessive use may undermine the physiological benefits of high-tech propagation environments, as demonstrated, who observed stress-related morphological anomalies under high retardant pressure in greenhouse crops.

## 5. Conclusions

As commercial rosemary production continues to expand, particularly in water-limited and climate-sensitive regions, the integration of advanced propagation systems into sustainable practices becomes increasingly necessary.

This study demonstrated that the propagation system, growth regulator, and auxin formulation exert significant and interactive effects on the morphological and physiological development of *Salvia rosmarinus* cuttings. Among the systems evaluated, the float method provided the most favorable balance between shoot elongation and root development, while aeroponics promoted superior lateral root branching under specific conditions. Growth regulators such as daminozide, when applied at moderate concentrations, enhanced root formation and overall cutting quality, particularly under mist and float systems, whereas higher doses induced growth suppression, especially in aeroponic environments.

Powder-based IBA formulations outperformed gel types across most systems, suggesting enhanced bioavailability and consistency in application. The statistically significant three-way interactions between system, hormone, and regulator highlight the need for context-specific optimization in nursery protocols. These findings confirm that soilless propagation outcomes are not solely defined by individual inputs but by the synergy between biological and technological factors.

Importantly, this work bridges a critical gap in the literature by integrating environmental conditions, hormonal manipulation, and growth modulation in a unified experimental framework.

Future research should explore scalable, high-efficiency models for rosemary propagation in vertical and soilless farming systems. Integration of propagation protocols into advanced horticultural setups—such as vertical farms, hydroponic units, and closed-loop water systems—could revolutionize the aromatic plant industry. These innovations will contribute to sustainable crop production, higher propagation efficiency, and climate-resilient practices suited for both urban agriculture and global supply chains.

## Figures and Tables

**Table 1:** Experimental design: number of replicates per treatment combination (n = 30).

Growth Retardant	Hormone (n = 30 Each)		System					
			Mist		Float		Aeroponic	
Control	Rooton A	Root !T	30	30	30	30	30	30
Daminozide 1000 ppm	Rooton A	Root !T	30	30	30	30	30	30
Daminozide 2500 ppm	Rooton A	Root !T	30	30	30	30	30	30
Daminozide 5000 ppm	Rooton A	Root !T	30	30	30	30	30	30
Paclobutrazol 1 ppm	Rooton A	Root !T	30	30	30	30	30	30
Paclobutrazol 2 ppm	Rooton A	Root !T	30	30	30	30	30	30
Paclobutrazol 5 ppm	Rooton A	Root !T	30	30	30	30	30	30

**Table 2:** Three-way ANOVA results showing the effects of propagation system, growth retardant concentration, and hormone formulation on shoot height of *Salvia rosmarinus* cuttings (n = 30 per treatment). All main effects and interactions were statistically significant (*p* < 0.001), highlighting strong context-dependent responses. Note: *** *p* < 0.001, ** *p* < 0.01.

Source	Sum of Squares	df	F-Value	*p*-Value
System	3422.90	2	511.92	<0.001 ***
Retardant	2638.20	6	131.52	<0.001 ***
Hormone	120.71	1	36.11	<0.001 ***
System × retardant	1153.14	12	28.74	<0.001 ***
System × hormone	34.90	2	5.22	0.006 **
Retardant × hormone	160.93	6	8.02	<0.001 ***
System × retardant × hormone	132.89	12	3.31	<0.001 ***

**Table 3:** Tukey HSD test for mean shoot height (cm). Significant differences (*p* < 0.05) are shown as letter groupings. Treatments not sharing a letter differ significantly.

Treatment	Shoot Height (cm)	Group
Float + powder + control	18.8 ± 1.2	a
Mist + powder + control	17.9 ± 1.1	a
Mist + gel + daminozide 1000	15.5 ± 1.0	b
Aeroponics + powder + paclobutrazol 5	12.1 ± 0.9	c

**Table 4:** ANOVA results for root length, indicating significant main and interaction effects between system type, hormone application method, and growth retardant concentration. The highest root elongation was recorded in float systems combined with powder-based auxin and moderate daminozide dosage. Note: *** *p* < 0.001, ** *p* < 0.01.

Source	Sum of Squares	df	F-Value	*p*-Value
System	1156.00	2	226.62	<0.001 ***
Retardant	348.50	6	45.10	<0.001 ***
Hormone	49.23	1	8.73	0.004 **
System × retardant	237.15	12	12.56	<0.001 ***
System × hormone	33.78	2	6.33	0.007 **
Retardant × hormone	80.12	6	5.42	<0.001 ***
System × retardant × hormone	101.67	12	4.26	<0.001 ***

**Table 5:** Tukey HSD post hoc comparisons of mean root length (cm). Statistically distinct groups are identified by letter codes.

Treatment	Root Length (cm)	Group
Float + powder + daminozide 1000	14.2 ± 1.3	a
Mist + powder + control	13.8 ± 1.2	a
Mist + gel + daminozide 1000	12.5 ± 1.0	b
Aeroponics + powder + daminozide 5000	10.6 ± 0.9	c
Aeroponics + gel + paclobutrazol 5	9.1 ± 0.7	d

**Table 6:** ANOVA summary for number of lateral roots, showing strong interactive effects among propagation system, hormone type, and growth retardant. Mist systems with powder hormone and low-dose daminozide significantly increased root branching (*p* < 0.001). Note: *** *p* < 0.001, ** *p* < 0.01.

Source	Sum of Squares	df	F-Value	*p*-Value
System	953.20	2	113.61	<0.001 ***
Retardant	376.31	6	42.82	<0.001 ***
Hormone	47.56	1	18.55	<0.001 ***
System × retardant	210.02	12	21.91	<0.001 ***
System × hormone	28.34	2	7.16	0.002 **
Retardant × hormone	39.60	6	5.89	<0.001 ***
System × retardant × hormone	59.87	12	3.98	<0.001 ***

**Table 7:** Tukey HSD comparison of root branching. Statistically significant groupings are marked with letters (*p* < 0.05).

Treatment	Lateral Roots (n)	Group
Mist + powder + daminozide 1000	51 ± 3	a
Float + powder + control	48 ± 2	a
Mist + gel + control	43 ± 3	b
Aeroponics + gel + daminozide 1000	39 ± 3	c
Aeroponics + gel + paclobutrazol 5	35 ± 2	d

**Table 8:** Mean shoot height (cm) at 0 and 60 days post-transplant.

System	Height at 0 Days (cm)	Height at 60 Days (cm)
Aeroponics	13.6 ± 2.1	21.7 ± 2.1
Mist	12.9 ± 1.4	20.9 ± 1.5
Float	12.0 ± 1.4	18.9 ± 1.5

## Data Availability

The data presented in this study are available on request from the corresponding author G.L.

## Supplementary Materials

The following supporting information can be downloaded at: https://www.mdpi.com/article/10.3390/plants14142210/s1. Table S1: Summary of physiological and morphological traits of Viola tricolor seedlings under varying temperature and nutrient treatments.
